# An Assessment of the Impact of Flow Disruptions on Mental Workload and Performance of Surgeons During Percutaneous Nephrolithotomy

**DOI:** 10.7759/cureus.14472

**Published:** 2021-04-13

**Authors:** Sana Hussain, Syed M Nazim, Basit Salam, Nida Zahid, M Hammad Ather

**Affiliations:** 1 Surgery, Aga Khan University Hospital, Karachi, PAK; 2 Radiology, Aga Khan University Hospital, Karachi, PAK; 3 Epidemiology and Public Health, Aga Khan University Hospital, Karachi, PAK; 4 Section of Urology, Department of Surgery, Aga Khan University Hospital, Karachi, PAK

**Keywords:** percutaneous nephrolithotomy, endourology, stone disease, urolithiasis, distractions, teamwork, surgeons' workload, interruptions, operation theater, work environment

## Abstract

Objective

The aim of this study was to assess the impact of intraoperative disruptions on surgeons’ workload and performance during percutaneous nephrolithotomy (PCNL).

Materials and methods

A structured and standardized tool was used to identify disruptions and interferences that occurred during 33 PCNL procedures. The surgical steps during PCNL were divided into four phases: ureteric catheter placement (phase I), puncture and tract dilation (phase II), intra-calyceal navigation and stone fragmentation (phase III), and tube placement (phase IV). Surgeons’ workload was evaluated using a validated tool: Surgery Task Load Index (SURG-TLX), and correlated with the mean observed intraoperative disruptions. All operating team members evaluated the teamwork immediately after the procedure. Statistical analysis was performed using SPSS Statistics version 22 (IBM, Armonk, NY).

Results

A total of 1,897 disturbances were observed, with an average of 57.48 ± 16.36 disruptions per case. The largest number of disruptions occurred during phase III of PCNL (32.06 ± 14.12). The most common cause of the disruption was people entering or exiting the operating room (OR) (29.1 ± 10.03/case), followed by the ringing of phones or pagers (6.42 ± 2.4). The mean observed intraoperative disruptions were significantly associated with the operating surgeon’s mental workload, and it had a significant impact on all domains of surgeons’ mental workload as measured by SURG-TLX. Compared to other team members, surgeons’ assistants experienced an inferior sense of teamwork (r=-0.433; p=0.012).

Conclusion

Significant intraoperative disruptions were observed during PCNL. They were observed to directly correlate with the surgeon's workload and had a detrimental effect on teamwork. Improving OR dynamics by reducing unnecessary disruptions would help establish an efficient and smooth surgical work environment for safe surgical care.

## Introduction

Working in the operating room (OR) comes with its own set of psychological and physical stressors. A surgeon's ability to perform a safe procedure is significantly influenced by efficient teamwork and minimal OR disruptions. Disruptions in the OR environment may add to mental stress and adversely affect surgical performance and outcomes. Leape et al. defined medical error as an unintended act, either of omission or commission, or one that does not achieve its intended outcome [1].

Medical error is the third most common cause of death in the US [2]. Surgical disciplines account for many of these errors, and up to two-thirds of these adverse effects are related to a surgical procedure [3]. In view of the potential of significant complications during surgery, it is imperative that the work environment in the OR should be a peaceful and organized one. Disruptions during surgical procedures have many sources. These include the ringing of cell phones and pagers; people entering or exiting OR; and the presence of trainees and students. These disturbances can potentially affect surgeons’ perceived workload, teamwork, and surgical outcomes. Weber et al. [4] quantified intraoperative disruptions and their impact on surgeons’ workload and perioperative teamwork and noted that intraoperative disruptions occur frequently and are associated with an increased mental workload.

Percutaneous nephrolithotomy (PCNL) is a demanding endo-urological procedure and requires the undivided attention of the operating team. Like other percutaneous procedures, the margin of error in PCNL is narrow [5]. It is a complex procedure and has its own share of complications attributed to the surgical technique as well as the stone- and patient-related factors [6]. Smooth conduct of PCNL requires an interplay of various factors including the surgical team dynamics, which involves the scrub nurse, circulator, anesthesia team, and radiographer. 

To the best of our knowledge, this is the first study to be conducted to demonstrate the impact of intraoperative disruptions on surgeons’ perceived workload and OR teamwork during PCNL. We aim to identify these factors during PCNL and their relationship with the surgical team's performance.

## Materials and methods

This cross-sectional study was conducted in the OR of a university hospital from October 2019 to November 2020. The institutional review board approval was obtained (ERC # 2019-1192-3776) prior to the start of the study. A non-probability, consecutive sampling strategy was used for the inclusion of all PCNL cases performed during the study period.

The sample size was calculated with the aid of the PASS 2011 software (NCSS, LLC, East Kaysville, UT). The anticipated correlation between surgical intraoperative disruptions and the surgeon’s perceived workload was 0.49 [4], with a type 1 error of 5% and a power of 80%. The estimated sample size was 33 with a confidence interval of 10%.

The complexity of renal stones was determined by the Guy Stone Score (GSS) system [7]. We included all PCNL procedures for renal stones with a GSS of 1-3. Complete staghorn stones (GSS of 4), patients requiring bilateral PCNL, PCNL in a solitary kidney, and cases requiring ancillary procedures were excluded. The procedural steps were standardized and were reported in a study conducted at our institute in 2015 [7]. The surgical steps during PCNL were divided into four phases: ureteric catheter placement (phase I), puncture and tract dilation (phase II), intra-calyceal navigation and stone fragmentation (phase III), and tube placement (phase IV).

Operational definitions

Intraoperative Interruption

**Figure 1:**
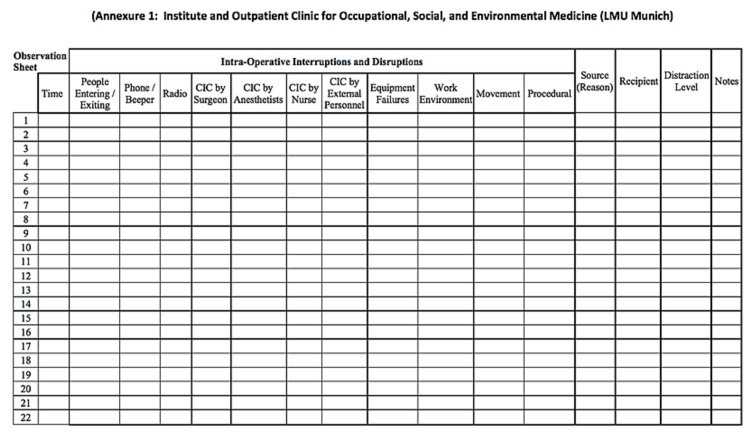
Observation tool CIC: case-irrelevant communication

“An event that deviates from the natural progression of the procedure, potentially distracts OR team members from their current task, or momentarily interrupts their task” [8]. An established tool to identify intraoperative workflow disruptions in the ORs was applied (Figure 1).

Surgical Workload

**Figure 2:**
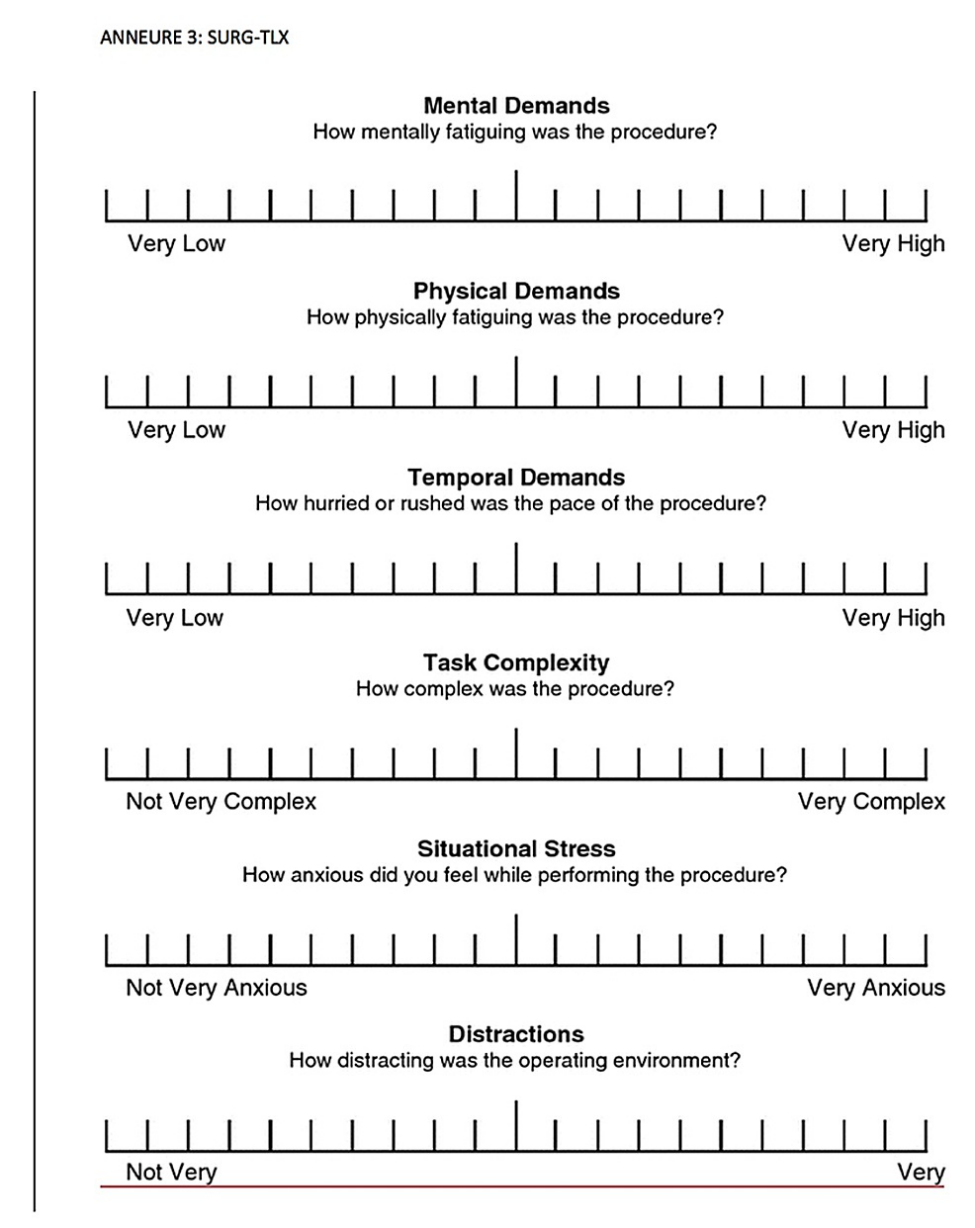
Surgery Task Load Index (SURG-TLX)

“The self-rated and perceived workload as measured by Surgery Task Load Index (SURG-TLX)” (Figure 2). The SURG-TLX is a multidimensional, surgery-specific workload measure, which involves six dimensions: mental demand, physical demand, temporal demand, situational stress, and distraction.

Teamwork

**Figure 3:**
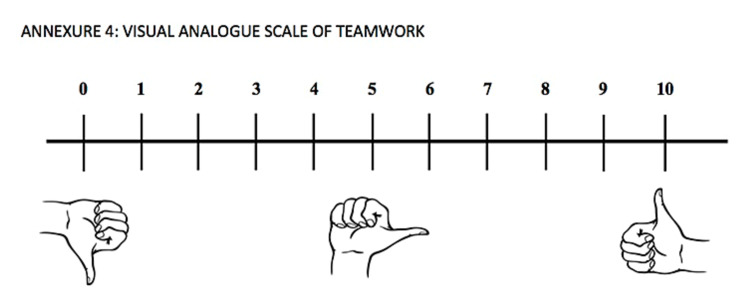
Teamwork visual analog scale

“Two or more people who interact interdependently with a common purpose, working toward measurable goals that benefit from leadership, maintains stability while encouraging honest discussion and problem-solving” [9]. Teamwork was measured on a visual analog scale ranging from 0 (very low) to 10 (very high) (Figure 3).

Data collection

The OR team members were informed about the study prior to the documentation of the observations. Written consent was obtained from all OR members on site. The study population comprised the urology OR staff including anesthetist, scrub nurse, scout nurse, surgeon, and surgeon's assistants involved in the surgery (PCNL). Patients' consents were not sought as patient identities and details were not gathered.

During each phase of PCNL (retrograde pyelography, access, lithotripsy, nephrostomy/stent placement), the number and type of disruptions were recorded by the investigators on the attached observation sheet with the permission of Weigl et al. (Figure 1). The primary surgeon was asked to rate surgical workload on SURG-TLX [10].

As mentioned above, SURG-TLX is a multidimensional, surgery-specific workload measure. It has six dimensions and each index was rated from 0 to 100 with an increment of 5 points, with 0 being very low and 100 being very high. The six dimensions are as follows: mental demand (“how mentally tiring was the procedure?”), physical demand (“how physically fatiguing was the procedure?”), temporal demand (“how hurried or rushed was the pace of the procedure?”), task complexity (“how complex was the procedure?”), situational stress (“how anxious did you feel while performing the procedure?”), and distraction (“how distracting was the operating environment?”) (Figure 2: SURG-TLX).

At the end of the procedure, each OR team member including the surgeon, assistants, scrub and circulating nurse, and the anesthetist were asked to answer the following question: “how would you rate the teamwork during the procedure?” The answers were assessed on a visual analog scale ranging from 0 (very low) to 10 (very high) (Figure 3).

Statistical analysis

All analyses were performed using SPSS Statistics version 22.0 (IBM, Armonk, NY). For descriptive statistics, we computed mean ± SD. Associations between intraoperative and surgeons’ perceived workload correlation analyses were performed, and Pearson’s correlation coefficient was assessed. A p-value of 0.05 was considered statistically significant.

## Results

Overall, 33 procedural observations were conducted; workload evaluations were collected from the operating surgeons. Teamwork evaluations were collected from the OR team members.

A total of 1,897 disruptions were noted. A mean of 57.48 ± 16.36 disruptions was recorded per case (Table 1). Most disruptions were noted during phase III/lithotripsy (mean: 32.06 ± 14.12), which is among the most demanding steps during PCNL (Table 2). Staff entering or exiting the OR (mean: 29.06/SD=10.028) was the most common disruption (Table 1). In addition, frequent disruptions due to phone or pager (6.42 ± 2.41), as well as case-irrelevant communications (CIC) by different people, were also observed frequently (Table 1).

**Table 1:** Type of interruptions observed during PCNL SD: standard deviation; OR: operating room; PCNL: percutaneous nephrolithotomy; CIC: case-irrelevant communication; PAS: announcements/voice pages

Interruptions	Mean	SD
People entering or exiting OR	29.06	10.028
Phone/pager	6.42	2.411
CIC by surgeon	3.88	3.150
CIC by anesthetist	3.59	2.312
CIC by nurse	5.00	3.905
External-person CIC	6.09	5.456
Equipment failure	1.00	1.436
Movement	0.64	1.319
PAS	1.21	1.474
Procedure	1.36	7.656
Work environment	0.64	1.884
Overall	57.48	16.363

**Table 2:** Phase-wise interruptions SD: standard deviation

	Overall interruptions	Phase I	Phase II	Phase III	Phase 4
Mean	57.48	8.64	10.15	32.06	6.61
SD	16.36	4.10	4.93	14.12	2.36

Intraoperative disruptions and surgeons’ workload

In order to identify the unique contribution of each of the intraoperative disruptions on surgeon's workload, we conducted partial correlation analyses. Statistical analysis of surgeons’ perceived workload revealed that the mean reported intraoperative disruptions were significantly associated with surgeons’ perceived workload as shown in Table 3. Mean intraoperative disruptions were associated with surgeons' reported workload: staff entering/exiting OR led to increased mental demand (r=0.52, p=0.002), physical demand (r=0.517, p=0.002), temporal demand (r=0.561, p=0.001), situational stress (r=0.542, p=0.001), and task complexity (r=0.42, p=0.014), while it only slightly increased surgeon's distractions (r=0.375, p=0.032). Furthermore, CIC by surgeons was associated with decreased distraction experienced by surgeons (r=-0.22, p=0.21), although it was not statistically significant. Table 3 also presents the associations between the overall summed disruptions and the workload indicators. Overall, the numbers of observed disruptions were significantly associated with surgeons' experienced distractions during the procedures.

Intraoperative disruptions affected perioperative teamwork in the OR, particularly among the group of surgeons’ assistants as shown in Table 4. However, there was a negative correlation of disruptions with teamwork. This was observed particularly among the group of surgeons’ assistants, who experienced an inferior level of teamwork in comparison to the rest of the team.

**Table 3:** Correlation of intraoperative interruptions with surgeons’ workload (Pearson’s correlation and p-values) *Correlation is significant at 0.05 level, using Pearson's correlation (two-tailed). **Correlation is significant at 0.01 level, using Pearson's correlation (two-tailed) SURG-TLX: Surgery Task Load Index; CIC: case-irrelevant communication; PAS: announcements/voice pages

Interruption	SURG-TLX, mental demand	SURG-TLX, physical demand	SURG-TLX, temporal demand	SURG-TLX, task complexity	SURG-TLX, situational stress	SURG-TLX, distractions
	Correlation (p-value)	Correlation (p-value)	Correlation (p-value)	Correlation (p-value)	Correlation (p-value)	Correlation (p-value)
People entering/exiting	0.526**(0.002)	0.517** (0.002)	0.561** (0.001)	0.424* (0.14)	0.542** (0.001)	0.375* (0.032)
Phone/pager	0.309 (0.080)	0.116 (0.519)	0.392* (0.024)	0.354* (0.043)	0.188 (0.296)	0.174 (0.332)
PAS	0.034 (0.850)	0.286 (0.107)	0.157 (0.384)	0.341 (0.052)	0.335 (0.057)	0.185 (0.302)
CIC - surgeon	0.169 (0.346)	-0.025 (0.891)	-0.041 (0.821)	-0.118 (0.512)	-0.186 (0.299)	-0.223 (0.212)
Equipment failure	-0.243 (0.173)	-0.136 (0.451)	-0.093 (0.606)	0.033 (0.854)	-0.275 (0.121)	-0.078 (0.667)
Work environment	-0.058 (0.748)	-0.041 (0.819)	0.020 (0.912)	0.163 (0.366)	0.066 (0.716)	0.160 (0.373)
Movement	0.022 (0.905)	0.030 (0.870)	0.232 (0.194)	0.293 (0.098)	0.094 (0.602)	0.181 (0.313)
Procedural	0.120 (0.504)	0.043 (0.813)	0.060 (0.739)	0.015 (0.934)	0.246 (0.167)	0.161 (0.370)
Overall interruptions	0.465** (0.006)	0.464** (0.007)	0.626** (0.000)	0.544** (0.001)	0.423* (0.014)	0.295 (0.096)

**Table 4:** Correlation between interruptions and teamwork *Correlation is significant at 0.05 level, using Pearson’s correlation (two-tailed) SD: standard deviation

Correlation between interruptions and teamwork					
Mean overall interruptions with mean overall teamwork by the surgical team					
Observed mean Interruptions=57.48 (SD=16.363)					
	Teamwork - surgeon	Teamwork - assistant	Teamwork - scrub nurse	Teamwork - circulator	Teamwork - anesthetist
Mean (SD)	7.12 (0.857)	7.88 (0.781)	8.70 (0.847)	8.70 (0.684)	7.82 (1.014)
Pearson’s correlation	-0.060	-0.433*	-0.223	-0.026	-0.113
P-value	(0.740)	(0.012)	(0.211)	(0.888)	(0.531)

## Discussion

This study aimed to investigate the disruptions during PCNL and correlate those with surgeons’ perceived workload and the effects on teamwork. A validated observation sheet was used to identify intraoperative workflow disturbances in the ORs [11].

The most frequently observed disruption in our study was the one encountered due to "OR traffic" in and out of the operating room. Intraoperative disruptions by staff entering or exiting the OR were the most frequently observed disruption in our study. In addition, frequent disruptions due to the ringing of the phone or pager as well as CICs are common in the healthcare settings [12], which is reflected in our study. A majority (80%) of the studies on the subject have noted noise to be detrimental to communication and surgical performance, particularly regarding total errors and the time to complete the task. Case-irrelevant verbal communication has been noted as the most common cause of disruption and is known to negatively affect patient care. Moreover, we have observed that the majority of disruptions were noted during phase III (lithotripsy), which is often the longest phase of PCNL, requiring surgeons’ intense attention, physical stability, and hand-eye coordination. There is a danger of fragmentation device-related mucosal injury resulting in mucosal bleeding and blurring of vision during this phase [13].

Our study demonstrated that intraoperative disruptions affect perioperative teamwork in the OR, particularly among the group of surgeons’ assistants. Previous work by Weigl et al. [8] has demonstrated that they experienced an inferior level of teamwork than nurses and anesthetists [14].

We also observed that surgeons’ intraoperative workload scores significantly correlated with the overall volume of disruptions, which is in line with the findings of previous studies [4,14]. This effect was particularly noted with staff entering or exiting OR, as well as phones and ringing pagers. Studies conducted in the ORs have shown that minor disruptions tend to collectively culminate in serious adverse events such as surgical errors [15]. An understanding of the nature and characteristics of these minor flow disruptions can help improve the overall safety of the operating procedure.

Surgeons’ attention and awareness are fundamental for decision making, clinical reasoning, and monitoring the rapidly evolving perioperative needs. In order to provide a safer OR for performing an uninterrupted surgical procedure, OR environments may need to be rid of unneeded disruptions. Of note, increased subjective distraction may impact attention or memory processes that are important in resuming previously interrupted tasks.

To the best of our knowledge, this is the first study to report the relationship of intraoperative disruptions and their correlation with surgeons' perceived workload and teamwork during PCNL using validated tools. Another strength of our study is the diversity of data, which was collected from the OR practice of multiple urologists at our center. This approach, however, could not capture the subjective or experienced interference in the OR. The duration of surgeries in our study was between two to three hours and, therefore, we could not capture other potential disruptions associated with longer-duration procedures, such as staff breaks and shift replacements. Expertise and individual coping strategies to deal with disruptions were not taken into account either. Since we observed cases with an anticipated duration of <3 hours, our findings could not simply be extrapolated to longer procedures that usually face further disruptions because of staff rotation or breaks, e.g., the rotating staff takes lunch breaks and is often replaced.

Although individual/team strategies to deal with these disruptions were not taken into account, we believe that feedback sessions and team training would be the best strategies to minimize these disruptions. Keeping pagers and cell phones on silent mode, and ensuring minimal verbal communication could be some measures that could help with the reduction in workflow disruptions. These measures will also be helpful in structuring interprofessional collaboration through better organization of various tasks. Overall, these would be promising strategies to optimize intraoperative procedural safety.

## Conclusions

Based on our findings, there is a significant association between intraoperative disruptions and surgeons’ perceived workload as well as teamwork by the OR team during PCNL. The surgery and surgeon both require undivided attention for a safe and effective surgical flow. Reducing mutual workflow disruptions and structuring interprofessional collaboration through better organization of various tasks is a promising strategy to optimize clinical safety.
